# Moving from stable standing to single-limb stance or an up-on-the-toes position: The importance of vision to dynamic balance control

**DOI:** 10.1371/journal.pone.0307365

**Published:** 2024-07-23

**Authors:** John G. Buckley, Sorcha-Sinead Frost, Shaun Hartley, Andre L. F. Rodacki, Brendan T. Barrett

**Affiliations:** 1 School of Engineering, University of Bradford, Bradford, United Kingdom; 2 Department of Physical Education, Federal University of Paraná, Curitiba, Paraná, Brazil; 3 School of Optometry and Vision Science, University of Bradford, Bradford, United Kingdom; University of Giessen: Justus-Liebig-Universitat Giessen, GERMANY

## Abstract

Understanding the contribution vision has to dynamic balance control may help in understanding where/why loss of balance occurs during everyday locomotion. The current study determined how body-centre-of-mass (BCoM) dynamics and postural stability when moving to and holding a single-limb-stance (SS) or an up-on-the-toes (UTT) position were affected by visual occlusion. From standing on a force platform, 18 adults (mean (SD) 26.7 (4.8) years; 1.73 (0.08) m; 84.0 (22.9) kg; 7 females) completed repeated trials (x3) with and without vision in which they moved to either a SS or an UTT position (order countered-balanced), and attempted to hold that position for 2 (SS) or 5 (UTT) seconds before returning to standing. UTT trials were also repeated at a fast speed, and SS trials were repeated using both the dominant and non-dominant limb. BCoM dynamics were assessed by analysing the displacement and peak velocity of the centre-of-pressure (CoP) when moving to and from the SS and UTT positions. Balance stability was the variability in the CoP displacement/velocity when holding these positions. Results indicate that under visual occlusion, the peak CoP velocity when moving to the SS or UTT position was reduced (ES, 0.67 and 0.68, respectively), suggesting greater caution. Both the variability in the CoP displacement/velocity when holding these positions and the peak CoP velocity when returning to flat-standing increased (SS: ES, 1.0 and 0.86, respectively; UTT: ES 1.26 and 0.66, respectively), suggesting, respectively, greater instability and poorer control. The poorer control in SS trials, occurred when returning to standing from the SS position held on the non-dominant limb, and correspondingly, the reduction in SS duration when vision was occluded was greater for the non-dominant limb trails (limb-vision interaction; p = 0.042). This suggests that movements initiated/controlled by the non-dominant limb are more reliant on visual feedback than those initiated/controlled by the dominant limb.

## Introduction

Control of standing posture and stability is fundamental to daily living and is a prerequisite (i.e., the starting position) for many whole-body locomotive movements. Initiating any whole-body locomotive-type movement involves moving from the relatively stable condition of bipedal standing to a relatively less stable state. This relatively less stable state often involves bodyweight momentarily becoming supported on a single limb as it moves forwards, sideways, and/or up-and-down, as when stepping forwards or up- or downwards, or during the everyday task of dressing when putting on trousers, socks and shoes. Moving to a relatively less stable state can also involve bodyweight momentarily becoming supported on a reduced base of support, such as when rising onto the toes when reaching for an item at an elevated position.

Moving from the relatively stable standing condition to a relatively less stable, dynamic state requires remarkable coordination. For example, before lifting the leading foot off the ground to initiate a step, the bodyweight must be shifted onto the contralateral limb first. Controlling the excursion of the BCoM when initiating a step is crucial because there is potential for a sideways fall over the contralateral limb if the sideways ‘shift’ imparted to the BCoM is too great. Conversely, too small a ‘shift’ would result in the BCoM quickly falling back towards the swinging limb, limiting the time to swing the limb forward, and so resulting in a shorter or stuttered step. Similarly, when rising onto the toes/balls of the feet (involving dorsiflexion at the metatarsophalangeal joint), a sufficient upward ‘shift’ of the BCoM must be generated to ensure that bodyweight (and thus the BCoM) is moved upwards and forwards onto the ball/toes area of the feet. Too small a ‘shift’ would mean the BCoM does not rise far enough for it to move onto the balls of the feet, resulting in a swift return to flatfoot standing. Too great a ‘shift’ would likely result in the BCoM moving onto the tips of the toes, which would be difficult to attain balance at and could result in toppling forwards. The above suggests that the successful initiation of such whole-body movements relies on the neuromuscular system imparting the precise amount of ‘shift’ to the BCoM to produce the appropriate amount of BCoM movement.

Shifting the BCoM onto a single limb is a prerequisite for gait initiation [1–3]. Previous research has shown that age can affect the contribution vision has to this task [4]. The study demonstrated that visual deprivation in older adults led to a significant reduction of forward trunk accelerations during the anticipatory phase of stepping, which resulted in a significantly reduced first step length and velocity and prolonged duration of the first step. In contrast, young adults did not respond to absence of vision by significant changes of neither APAs, nor first step kinematics [4]. What is perhaps more germane to the current study is the work exploring BCoM dynamics when making a single step onto a foot-shaped target placed on the floor in front of a participant [5–7]. In such conditions, the shift imparted to the BCoM is related to the target’s relative position and how quickly the step is executed [6]. Hence, when making repeated steps at a set speed, the BCoM acquires greater forward velocity for steps made further forward and greater lateral velocity for steps made more laterally (i.e., away from the stance foot [5]). The shift imparted to the BCoM is noticeably variable when making repeated steps to such pre-defined locations, but the resulting foot placement precision is relatively high (i.e., low variability [7]). This suggests that vision is typically used after foot-lift in adjusting the swinging (stepping) limb when making targeted steps.

Standing postural control and stability can be evaluated by investigating the changes in the horizontal location of the point of application of the reaction force vector coming from the ground [8]. Ground reaction forces (GRFs) are measured using force platforms, and where the resultant force vector is located at any instant in time is determined as the centre of pressure (CoP). Hence, standing postural control and/or stability can be readily evaluated by monitoring the dynamically changing position of and/or fluctuations in the CoP while a person stands on a force platform. Here we describe instability as being associated with an increase in the variability of the CoP during standing [9]. A force platform can also be used to determine how whole-body movements are initiated by determining shifts in the CoP and the velocity of such shifts [10, 11]. These shifts reflect how the GRF is being directed to bring about changes in the BCoM position and its velocity, i.e., they reflect BCoM dynamics.

There has been some recent work assessing the importance of vision to BCoM dynamics when moving from bi-pedal to single-limb stance [12] and when moving from flatfoot standing to being up on the toes [13] but this work was conducted in girls aged 11 [13] or 16 years old [12]. Their findings indicate that when moving to a single-limb stance, the peak lateral CoP velocity (just over 500 mm/s) is reduced by around 11% when vision is occluded [12]. Similarly, when moving onto the toes, the peak forward CoP velocity (just under 500 mm/s) is reduced by around 27% when vision is occluded [13]. In both studies, it was also found that postural stability when holding the single-limb stance or up-on-the-toes position became diminished (increased instability) when vision was occluded. However, given the population studied, these results may not necessarily represent what happens in mature adults. For example, in the study investigating moving to a single-limb position [12], it was found that limb dominance (dominant versus non-dominant) had no effect on BCoM dynamics, irrespective of vision condition. This may highlight that limb dominance in children is not as important as in mature adults.

The current study aimed to determine if and how the BCoM dynamics and postural stability when moving to, and then maintaining, a single-limb stance and an up-on-the-toes position are affected by visual occlusion in mature adults. It also aimed to determine the importance of vision to the control of BCoM dynamics when moving from these temporarily held positions back to bipedal flatfoot standing. Additionally, we sought to determine if visual occlusion affects moving to a single-limb stance position differently for the dominant versus non-dominant limb and affects moving to an up-on-the-toes position differently if the movement is completed as fast as possible. It is important to understand the influence of vision on the BCoM dynamics and postural stability when initiating everyday whole-body locomotive movements, as it provides insight into the contribution of vision to dynamic balance more generally. Understanding how vision influences dynamic balance may help in understanding where and why loss of balance occurs during everyday locomotion, especially when severe or less severe visual impairments are present. Rather than investigate the effects of visual impairment (e.g., by blurring or partly occluding vision), we investigate the effects of fully occluding vision as a first step in identifying the contribution that vision might make to the execution of these tasks. If occluding vision has no impact, then there would be little point in studying if less dramatic degradations of vision (e.g., blurring, restriction of the visual field) impacted the execution of these tasks.

## Materials and methods

### Participants

Recruitment of participants began 14^th^, November 2022 and ended on 20^th^, January 2023. An opportunist sample of eighteen healthy young adults (mean (SD) 26.7 (4.8) years; 1.73 (0.08) m; 84.0 (22.9) kg; 7 females) agreed to participate in the study. Sixteen indicated they were right-foot dominant (e.g., the chosen limb to kick a football), and two confirmed they were left-foot dominant. Any participant who habitually used corrective spectacles or contact lenses did so during data collection. All participants self-reported they were visually normal (i.e., no amblyopia and good vision in each eye). Before data collection, all gave written informed consent. Ethical approval was obtained from the institutional bioethics research committee, and the tenets of the Declaration of Helsinki were observed.

### Protocol

Participants stood in a standardised position: feet side-by-side and apart by 11% of the stature, with their feet externally rotated by 14° [14], on a force platform (AMTI OR6-7) with their gaze directed to a large, easily visible “X”, placed at 4.3m ahead, at eye level. They stood with their arms crossed over their chest and were barefoot. After approximately 3 s of quiet standing (which established a stable baseline of the ground reaction forces (GRF) and centre of pressure (CoP) coordinates), participants were asked to either (1) “rise” up onto their toes (UTT, block 1) or (2) move to a single-limb supported (SS) position (block 2), and attempt to hold the position until instructed to return “down” to flat-foot standing, which was given after either 2 seconds (SS trials) or 5 seconds (UTT trials). Trials in both blocks were undertaken with and without visual occlusion, with vision occluded using a blindfold which prevented any light from reaching the eyes. The order in which each block was undertaken first (block 1 or 2) was counter-balanced across participants. The timings for when the verbal instructions were given were based on the forceplatform’s software recording clock, which gave a live ‘recording time’ when set to record. The 2 and 5 second timings were decided after conducting pilot work, which indicated that when vision was occluded standing on one limb was very difficult to maintain for more than 2 seconds, whilst standing up-on-the-toes under visual occluded condition could be maintained for 5 seconds. UTT trials were undertaken with participants rising up on their toes at a customary speed and as fast as possible. However, it quickly became clear that participants were unable to hold/maintain a UTT position when rising on their toes as fast as possible if their vision was occluded. Thus, this condition was excluded from the protocol, and thus, visual occlusion trials were only undertaken for the customary speed. Trials involving moving to a SS supported position were undertaken at customary speed for both left and right directions (i.e., moving from a bipedal stance to either a left- or right- limb SS position). Trials for each condition were repeated 3 times in a pseudo-randomised order. All trial data were collected within a single session for each participant.

### Data acquisition and processing

Ground reaction force (GRF) data were collected at 100 Hz. GRF data were low-pass filtered (20 Hz cut-off) and used to determine the CoP (x and y) coordinates. Data were subsequently exported for further analysis. All outcome measures were determined for each trial using customized routines written in Visual Basic.

### Data analysis

**Fig 1:**
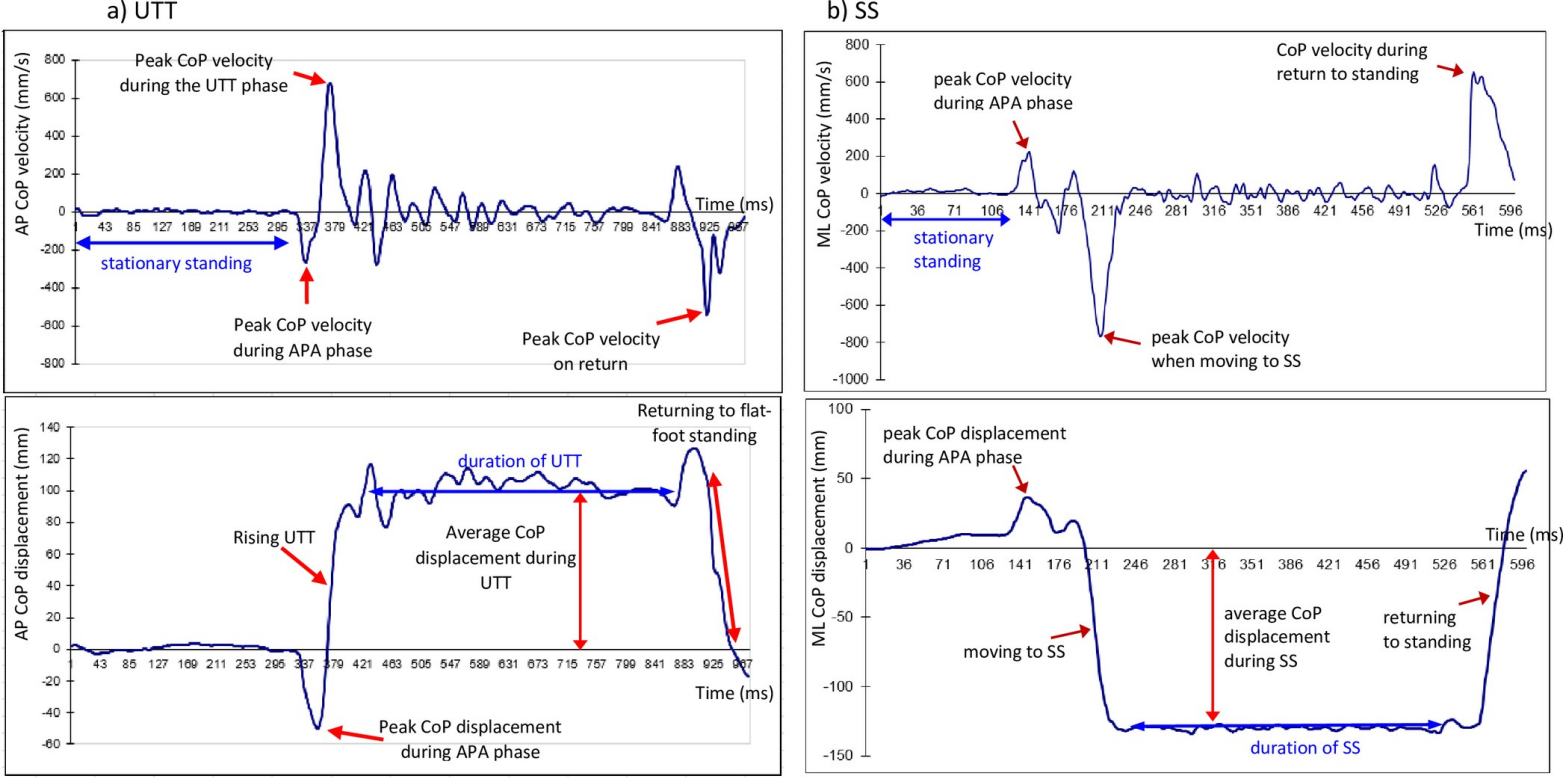
Definitions of the CoP variables determined: a) UTT task. CoPAP velocity (mm/s, upper left panel) and CoP displacement (mm, lower left panel) when rising from a stable standing position to an UTT position, holding it for 6 seconds and returning to standing, and b) SS task. the CoPML velocity (mm/s, upper right panel) and CoPML displacement (mm, lower right panel) for when moving from a stable standing position to SS position, holding it for 3 seconds and returning to standing (SS). The numbers on the x-axis of UTT figure correspond to frame number (each frame 0.01 seconds): the frames on the x-axis of the SS figure have been removed to aid clarity. The labels in the figures indicate the different parameters analysed (see Table 1 for details regarding how each outcome was determined. (Footnote) The anticipatory postural adjustment (APA) phase, is the period involving activation of postural muscles in a feedforward manner, in anticipation of the destabilizing forces caused by the ensuing voluntary movement.

The definitions of all the variables are presented in Table 1. These variables were determined by analysing the displacement and velocity of the CoP when moving to a UTT (Fig 1A) or SS (Fig 1B) position. As moving to a UTT position predominantly involves movement in the sagittal plane, CoP variables for the UTT task (Fig 1A) were computed in the anterior-posterior (AP) direction only. Similarly, as moving to an SS position predominantly involves movement in the frontal plane, CoP variables for the SS task (Fig 1B) were computed in the mediolateral (ML) direction only. The parameters (outcome measures) determined for each trial and every participant, across the two movement tasks (moving to up-on-the-toes [UTT] or to single-limb stance [SS] position) are included as (see S1 and S2 Files).

**Table 1:** The variables of the up-on-the toes (UTT) and Single-limb stance (SS) and their operational definition.

	phase	Variable	Definition
**UTT** (see Fig 1A)	APA	CoP_AP_ Displacement APA (DCoP_AP-APA_) (mm)	Peak displacement in CoP_AP_ in the countermovement direction during the anticipatory postural adjustment (APA) phase relative to the average CoP_AP_ displacement during stationary standing.
		Peak CoP_AP_ velocity APA (VCoP_AP-APA_) (mm.s^-1^)	Peak CoP_AP_ velocity in the countermovement direction during the APA phase.
	UTT period	Peak CoP_AP_ velocity UTT (VCoP_AP-MOV_) (mm.s^-1^)	Peak CoP_AP_ velocity when rising on to the toes.
		Time of peak CoP_AP_ velocity UTT (tVCoP_AP-UTT_) (s)	Time from movement initiation up to the instant of peak CoP_AP_ velocity during rising UTT. Movement initiation was determined as the instant where CoP displacement increased in the countermovement direction to <-15mm.
		Time to start of UTT (tStart_UTT_) (s)	The time from movement initiation to the start of the UTT period. The start of the UTT period was determined as the instant where CoP_AP_ velocity reduced to ≤150 mm/s following the instant of peak CoP_AP_ velocity UTT.
		Average CoP_AP_ displacement when UTT as % of foot-length (%DCoP_AP-UTT_) (%)	The average displacement of CoP_AP_ during the UTT period, relative to average CoP_AP_ displacement when standing stationary: recorded as a percentage of foot length.
		CoP_AP_ standard deviation UTT (SDCoP_AP-UTT_) (mm)	Standard deviation of CoP_AP_ during the UTT period.
	Return	Peak CoP_AP_ velocity return (RnVCoP_AP_) (mm.s^-1^)	Peak CoP_AP_ velocity in countermovement direction when returning to flat foot standing
**SS** (see Fig 1B)	APA	CoP_ML_ displacement APA (DCoP_ML-APA_) (mm)	Peak displacement in the CoP_ML_ in the countermovement direction during the APA phase relative to the average CoP_ML_ position during stationary standing.
		Peak CoP_ML_ velocity APA (VCoP_ML-APA_) (mm.s^-1^)	Peak CoP_ML_ velocity in the countermovement direction during the APA phase.
	SS period	Peak CoP_ML_ velocity moving to SS (VCoP_Ml-MOV_) (mm.s^-1^)	Peak CoP_ML_ velocity when moving to SS.
		Time start SS (tStart_SS_) (s)	The time from movement initiation to the start of the SS period.
		Average CoP_ML_ displacement when in SS (DCOP_ML-SS_) (mm)	The average CoP_ML_ displacement during the SS period relative to the average CoP_ML_ position when standing stationary.
		CoP_ML_ velocity standard deviation (SD-VCoP_ML-SS_) (mm.s^-1^)	The CoP_ML_ velocity standard deviation for the period of SS.
		Period of SS (SS_TIME_) (s)	The time from the start to end of SS. The end of SS was determined as the instant where CoP_ML_ velocity in the countermovement direction increased to >200 mm/s following the start of SS.
	Return	Peak CoP_ML_ velocity return (RnVCoP_ML_) (mm.s^-1^)	Peak CoP_ML_ velocity in the countermovement direction when returning to flat foot standing.

APA, anticipatory postural adjustment

### Statistical analysis

Data were analysed using Repeated Measures ANOVA with the experimental conditions as the repeated factor. The data were checked for normality; and the Shapiro-Wilk test confirmed data normality, and the Levene test confirmed homogeneous variances. For when moving to UTT, the “conditions” (customary speed with and without vision, fast with vision), were included as one repeated measure factor (i.e. 1-way ANOVA). When moving to an SS, the effect of limb (dominant vs. non-dominant) and vision (full vs. occluded) were included as repeated measure factors (i.e. 2-way ANOVA). Post-hoc analysis was undertaken using the Holm method [15]. All statistical analyses were undertaken using the JASP software (Version 0.18.1), and the significance level was set at p<0.05. Due to the exploratory nature of the study, no alpha corrections were made to account for multiple comparisons [16].

## Results

### Importance of vision when moving to and holding a UTT position and when returning to flat-foot standing

The results of the UTT test across experimental conditions are provided in Table 2.

**Table 2:** Effects of vision occlusion and speed of movement (customary versus fast as possible) on CoP_AP_ displacement and velocity measures and temporal characteristics of the UTT test.

Variable	Full vision/ Cust speed	Vision Occluded/ Cust speed	Full vision/ Fast speed	Condition main effect
DCoP_AP-APA_ (mm)	45 (10)	36 (12)*	63 (16)*^+^	**< 0.001**
VCoP_AP-APA_ (mm.s^-1^)	255 (78)	211 (65)^	413 (127)*^+^	**< 0.001**
VCoP_AP-UTT_ (mm.s^-1^)	657 (187)	511 (218)*	1076 (250)*^+^	**< 0.001**
%DCoP_AP-UTT_ (%)	37 (6)	35 (6)	36 (5)	= 0.206
SDCoP_AP-UTT_ (mm)	11 (3)	20 (7)*	11 (3)	**< 0.001**
RnVCoP_AP_ (mm.s^-1^)	416 (115)	529 (201)*	500 (170)	**= 0.046**
tVCoP_AP-UTT_ (s)	0.42 (0.08)	0.43 (0.11)	0.33 (0.06)*^+^	**< 0.001**
tSTART_UTT_ (s)	0.62 (0.09)	0.61 (0.11)	0.51 (0.08)*^+^	**< 0.001**

Post-hoc (Holm) comparisons

*significantly different to Full vision/Cust speed

^+^significantly different to Vision occluded/Cust speed

^difference trend (p = 0.066) compared to Full vision/Cust speed. Bold indicates statistically significant findings (p<0.05). APA, anticipatory postural adjustment.

During the anticipatory postural adjustment phase (APA), both the CoP_AP_ displacement (DCoP_AP-APA_) and the peak CoP_AP_ velocity (VCoP_AP-APA_) were greater for the fast compared to the customary speed trials (p<0.001, by 41% [ES, 1.16] and 62% [ES, 1.21] respectively). When vision was occluded, both variables were reduced, suggesting participants became cautious, but only the DCoP_AP-APA_ was significantly reduced (p = 0.004, reduced by 20% [ES, 0.76]), with the reduction in VCoP_AP-APA_ showing only a borderline trend (p = 0.066, reduced by 17% [ES, 0.59]. When moving UTT, the peak CoP_AP_ velocity (VCoP_AP-UTT_) was greater for the fast compared to the customary speed trials (p<0.001, by 64% [ES, 1.38]), and was reduced when vision was occluded (p = 0.006, by 22% [ES, 0.68]). The time from movement initiation to when peak CoP_AP_ velocity (tVCoP_AP-UTT_) and to when the UTT position was attained (tStart_UTT_) were unaffected by vision (p>0.48), but both times were shorter for the fast compared to customary speeds trials (p < .001, by 22% [ES, 1.08] and 17% [ES, 1.08], respectively).

When holding the UTT position, there was no difference in the average CoP_AP_ displacement-UTT (%DCoP_AP-UTT_) across speed or vision condition (p>0.21). The CoP_AP_ standard deviation UTT (SDCoP_AP-UTT_) was also unaffected by speed (p>0.95), but increased, indicating greater postural instability when vision was occluded (p<0.001, by 82% [ES, 1.26]). When returning to the flat-foot standing position, the peak CoP_AP_ velocity (RnVCoP_AP_) was unaffected by speed (p>0.145), but it increased (by 27%), suggesting poor control, when vision was occluded, although the significance level was borderline (p = 0.052, ES, 0.66).

### The importance of vision when moving to, and holding, a SS position and when returning to a flat-foot position

The results of the SS test across experimental conditions are provided in Table 3.

**Table 3:** Effects of vision occlusion and dominance on CoP_ML_ displacement and velocity measures, and temporal characteristics of the SS test.

	Full vision/ Dom	Occluded/ Dom	Full vision/ Non-Dom	Occluded/ Non-Dom	Main effects	Limb-vision interact
DCOP_ML-APA_ (mm)	57 (14)	52 (14)	59 (14)	52 (14)	Limb = 0.731 Vision = 0.053	= 0.747
VCoP_ML-APA_ (mm.s^-1^)	350 (116)	338 (113)	371 (97)	334 (79)	Limb = 0.558 Vision = 0.202	= 0.379
IMP_Ml-MOV_ (Ns)	26 (9)	23 (9)	27 (10)	25 (9)	Limb = 0.108 **Vision <0.001**	= 0.826
VCoP_ML-MOV_ (mm.s^-1^)	828 (164)	688 (154)	812 (212)	708 (160)	Limb = 0.944 **Vision <0.001**	= 0.483
DCoP_ML-SS_ (mm)	140 (19)	131 (21)	139 (12)	132 (12)	Limb = 0.962 **Vision <0.001**	= 0.702
SD-VCoP_ML-SS_ (mm.s^-1^)	131 (29)	169 (34)	121 (23)	176 (65)	Limb = 0.891 **Vision <0.001**	= 0.376
RnVCoP_ML_ (mm.s^-1^)	800 (410)	1100 (354)^	754 (356)	1327 (662)^+^	Limb = 0.311 **Vision <0.001**	= 0.168
tSTART_SS_ (s)	0.77 (0.06)	0.79 (0.11)	0.77 (0.12)	0.85 (0.16)^+^	Limb = 0.344 **Vision = 0.004**	= 0.172
SS_TIME_ (s)	3.25 (0.37)	2.96 (0.43)*	3.34 (0.30)	2.74 (0.43)^+^^#^	Limb = 0.376 **Vision <0.001**	= **0.042**

Post-hoc (Holm) comparisons

*significantly different to Full vision/ Dom

+significantly different to Full vision/ Non-Dom

^difference trend (p = 0.061) compared to Full vision/ Dom

#difference trend (p = 0.067) compared to Occluded vision/ Dom. APA, anticipatory postural adjustment.

During the APA phase, the peak CoP_ML_ velocity (VCoP_ML-APA_) was unaffected by limb (p = 0.56) or by vision condition (p = 0.20). Whereas the peak CoP_ML_ displacement (DCOP_ML-APA_), which was also unaffected by limb condition (p = 0.73), became reduced (by 10%), suggesting participants became cautious, under visual occlusion, though with borderline significance (p = 0.053, ES, 0.46). There were no interactions between terms (p>0.38).

When moving to the SS position, the peak CoP_ML_ velocity (VCoP_ML-MOV_) (p = 0.94) was unaffected by which limb the participant was standing on, but it became significantly reduced, again suggesting increased caution, when vision was occluded (p<0.001, by 15% [ES, 0.67]). There were no interactions between terms (p>0.38). The average CoP_ML_ displacement when holding the SS position (DCoP_ML-SS_) was unaffected by limb condition (p = 0.96), but it was reduced (by 6%), when vision was occluded (p<0.001, ES, 0.47). There were no interactions between terms (p>0.70). The time taken to attain the SS position (tStart_SS_) was unaffected by limb (p = 0.34) but increased (by 6%) when vision was occluded (p = 0.004, ES, 0.41). There was a weak trend interaction between limb and vision (p>0.17), and post-doc analysis indicated occlusion of vision had no effect on the tStartSS in dominant limb trials (p = 0.91), but it led to an increase (by 9%) in tStartSS in the non-dominant limb trials (p = 0.017).

When holding the SS position, there was no difference in the CoP_ML_ velocity variability (SD-VCoP_ML-SS_) across limb conditions (p = 0.89), but this measure increased (by 37%) when vision was occluded (p<0.001, ES, 1.0), indicating greater postural instability. There were no interactions between terms (p>0.38). The time holding the SS position (SS_TIME_) was unaffected by limb (p = 0.38), but it was significantly shorter (by 14%) when vision was occluded (p<0.001, ES, 1.0). In addition, a limb-by-vision interaction (p = 0.042) indicated the reduction in SS_TIME_ was greater for the non-dominant (18%) compared to the dominant limb (9%) trials.

When returning to flat-foot standing, there was no difference in the peak CoP_ML_ velocity (RnVCoP_ML_) across limb condition (p = 0.31), but it increased for both limbs (by on average 56%) when vision was occluded (p<0.001, ES, 0.86), indicating less control. There was an interaction trend between limb and vision (p>0.17), and post-doc analysis indicated occlusion of vision had a weak effect on the RnVCoP_ML_ in the dominant limb trials (p = 0.061) but led to an increase in RnVCoP_ML_ in the non-dominant limb trials (p<0.001).

## Discussion

The present study sought to determine the importance of vision to BCoM dynamics and postural stability when moving from a stable standing position to either a single-limb stance (SS) or an up-on-the-toes (UTT) position, holding the position for either 2 seconds (SS trials) or 5 seconds (UTT trials), and then returning to stable standing. The study determined whether, in mature adults, the BCoM dynamics and postural stability when moving to and then holding these positions are affected by visual occlusion in the same way as that recently reported for young girls [12, 13]. The occlusion of vision had a significant effect on 5 out of 8, and on 7 out of 8 variables analysed in the UTT and SS tests, respectively. Our results indicate that when visual feedback is unavailable, these everyday, whole-body locomotive movements were executed seemingly with more caution, and held with increased instability. These findings are in good agreement with the findings of Blaszczyk and colleagues that highlighted visual feedback is important in initiating everyday whole-body movements [12, 13].

The present study advances the work of Blaszczyk and colleagues because it also determined the importance of vision to the control of BCoM dynamics when moving from the temporarily-held positions of being UTT or being in SS, back to bipedal flat-foot standing. It also determined if visual occlusion affects moving to an SS position differently for the dominant versus the non-dominant limb, or affects moving to a UTT position differently if the movement is completed as fast as possible. The results indicate that visual occlusion led to a relatively large increase in the CoP_AP_ velocity (by ~27%) or in the CoP_ML_ velocity (by 38% and 76% for the dominant limb and non-dominant limbs, respectively) when returning to flat-foot / bipedal standing. This highlights that returning to flat-foot standing occurred more rapidly when visual information was unavailable, suggesting poorer control over the BCoM as would occur by simply dropping to flat-foot/bipedal standing, rather than being gradually lowered in a controlled manner. Interestingly, the lack of control over the BCoM under visually-occluded conditions was only significant when returning to flat-foot standing from an SS position in trials in which the SS position was held on the non-dominant limb. Correspondingly, the reduction in the time holding the SS position when vision was occluded was greater for the non-dominant (18%), in comparison to the dominant limb (9%) (i.e., a limb-vision interaction existed; p = 0.042). This suggests that the control of BCoM dynamics for movements initiated/controlled by the non-dominant limb is more reliant on visual feedback than those initiated/controlled by the dominant limb. This may be a consequence of differences in the neural control of the dominant limb compared to the non-dominant limb [17–19].

Although the BCoM dynamics (i.e., CoP_AP_ velocity) were seen to increase when rising to the UTT position at the fast compared to normal speed (Table 1), there was no difference across speed conditions in the average CoP_AP_ displacement or in the CoP_AP_ displacement variability when holding the UTT position. It was initially planned for participants to undertake the fast-speed trials both with and without vision occlusion. However, after testing the first few participants, it became apparent that they all were unable to hold the UTT position when completing the task at the fast speed if their vision was occluded. In these circumstances, all participants lost balance (i.e., by toppling forwards) immediately after rising onto their toes. Thus, this condition (fast, with vision occluded) was removed from the protocol. It appears that obtaining a UTT position after rising UTT as fast as possible becomes impossible for most participants if visual feedback is eliminated. Rising UTT as fast as possible generates a relatively large BCoM upward momentum that must be subsequently dissipated to establish a stationary, ‘stable’ UTT position. In effect, this upward momentum caused an undue balance perturbation, which could not be countered. It would be interesting to determine if and how degraded vision affects the control the BCoM dynamics when rising UTT at a fast speed.

### Implications of findings

Previous research reporting the importance of vision in postural control has classically investigated how bipedal standing is affected by the occlusion or degradation of vision [9, 20–24]. Previous studies have shown a larger dependence on visual feedback for standing balance control in older adults compared to their younger peers. The greater difficulties in sustaining or regaining stability have been related to the natural senescent decline in several sensory systems [25–30]. The greater dependence on visual feedback for standing balance control is more evident if older adults have their vision optimally corrected (e.g., by ensuring their refractive error is optimally corrected with spectacle or contact lenses) so that they gain the clearest possible visual information [20, 31, 32]. Other sensorial systems cannot be so readily ‘upgraded’ to provide improved feedback about postural stability. Studies of individuals who have experienced a major change to one sensory system provide information which is relevant to our findings [33–36]. For example, the visual feedback contribution to standing balance control and improved stability seems to be more critical in those with a lower-limb amputation, which is due to the disrupted somatosensory feedback from the residual limb [37, 38]. Interestingly, the large visual dependence in amputees is reduced through rehabilitation as they learn to integrate somatosensory feedback information and establish new neural pathways from the residual limb [38]. This may further emphasize the argument that due to poorer sensorial information from the non-dominant limb, more reliance is placed on visual feedback in controlling BCoM dynamics and stability for movements initiated/performed by the non-dominant limb. It may also be related to the fact that the dominant side tends to develop greater strength, coordination, and dexterity compared to the non-dominant side [17, 39].

The previous research, highlighted above, has provided valued insights into the importance of vision to standing postural control and stability, and how such might be different in different clinical populations. The present study has shown that vision occlusion has a similar impact on dynamic postural control (i.e., on BCoM dynamics and postural stability) when moving from stable standing to either a UTT or an SS position. These are basic, whole-body movements in everyday locomotion and/or everyday living. Future research should determine if the importance of vision to BCoM dynamics and postural stability, when moving from stable standing to either a UTT or an SS position, is different in patients with a history of falling, patients with reduced visual function, patients in whom there is altered proprioception in the feet (e.g., due to diabetic foot disease) or large differences in strength (asymmetry) between limbs. Such work could help in further understanding where and why loss of balance occurs in these patient groups who are known to have an increased prevalence of falling [25, 27, 40–42].

Recent work has shown that one-legged balance performance is a strong predictor of injurious falls [43] or of all-cause mortality among older adults [44]. However, in both of these studies, having good vision was not included as an assessed variable. The findings of the present study highlight the importance of vision to the BCoM dynamics and postural stability when moving to a single-leg stance (i.e., to a SS position). Hence, future work is required to investigate whether older adults, all of whom are likely to have age-related visual impairment, can move to and hold a SS position more proficiently when their vision is optimally corrected compared to their peers with uncorrected, or sub-optimally corrected vision. If this is found to be the case, it could suggest that performing a one-legged test may have an improved ability to predict injurious fall risk or all-cause mortality if it is performed without optimally-corrected vision.

## Conclusion

The results indicate that under visual occlusion, the peak CoP velocity when moving to the single stance or up-on-the-toes position became reduced, suggesting that participants became more cautious. In addition, both the variability in the CoP displacement/velocity when holding these positions and the peak CoP velocity when returning to flat-standing became increased, suggesting, respectively, an increase in instability and poorer control of BCoM dynamics during return to flat-foot/bipedal standing. These findings highlight that these whole-body movements, which are fundamental to everyday locomotion, can be completed with enhanced control over BCoM dynamics and with better postural stability when visual feedback is available. Finally, the poorer control in SS trials, occurred when returning to standing from the SS position held on the non-dominant limb, and correspondingly, the reduction in SS duration when vision was occluded was greater for the non-dominant limb trails. This suggests that movements initiated/controlled by the non-dominant limb are more reliant on visual feedback than those initiated/controlled by the dominant limb.

## Supporting information

S1 File Data all trials UTT. (PDF)

S2 File Data all trials SS. (PDF)
