# Supplementary material for: Moving from stable standing to single-limb stance or an up-on-the-toes position: The importance of vision to dynamic balance control
Source: PLoS One. 2024 Jul 23;19(7):e0307365. doi: 10.1371/journal.pone.0307365 (PMC11265682; doi:10.1371/journal.pone.0307365)
Supplement: S1 File — (PDF) [file pone.0307365.s001.pdf]

		CoP-dispA	CoP-vel Pk	Pk CoP-ve	CoP-SD SS	averageCO	Pk CoP-ve
P1	customNV1.xls	-30.11	-321.31	534.14	16.62205	82.93312	-295.19
P1	customNV2.xls	-48.4029	-283.23	666.63	16.03365	82.23292	-521.28
P1	customNV3.xls	-55.7765	-274.64	590.3	22.39685	74.48092	-401.58
P2	customNV1.xls	-41.3881	-95.93	380.95	18.31195	84.99164	-430.32
P2	customNV2.xls	-26.4924	-143.67	306.56	20.71295	107.4407	
P2	customNV3.xls	-21.26	-109.01	319.43	13.33757	88.6017	-288.21
P3	customNV1.xls	-27.2863	-119.24	294.31	9.933001	93.68849	-284.05
P3	customNV2.xls	-28.5601	-190.66	478.7	15.85151	74.74823	-666.9
P3	customNV3.xls	-15.2826	-86.92	413.25	15.88973	93.64705	-740.54
P4	customNV1.xls	-50.8409	-289.71	553.32	15.69892	87.35078	-821.59
P4	customNV2.xls	-53.6422	-300.07	723.51	14.23412	45.02842	-1264.84
P4	customNV3.xls	-31.6285	-111.98	589.43	12.3849	119.7751	-483.21
P5	customNV1.xls	-39.181	-197.78	373.6	16.16639	107.9963	-261.55
P5	customNV2.xls	-50.6145	-290.16	535.33	19.48871	118.72	-221.9
P5	customNV3.xls	-40.8925	-190.15	493.73	12.13886	98.68764	-340.12
P6	customNV1.xls						
P6	customNV2.xls	-16.811	-132	170.6	15.84337	54.55421	-543.15
P6	customNV3.xls	-29.49	-214.76	265.1	19.78721	51.95091	-1056.09
P7	customNV1.xls	-16.2837	-83.26	328.31	13.86686	89.47794	-391.5
P7	customNV2.xls	-22.3103	-172.43	197.9	19.28247	88.11849	-473.15
P7	customNV3.xls	-35.2052	-197.33	424.64	17.09304	95.58354	-309.23
P8	customNV1.xls	-64.5446	-315.61	997.31	24.87718	96.25599	-484.78
P8	customNV2.xls	-30.7046	-28.54	980.02	23.29529	123.8623	-290.48
P8	customNV3.xls	-54.861	-331.1	943.8	22.34369	133.1176	-747.95
P9	customNV1.xls	-15.0352	-79.63	244.89	25.12461	101.0592	-218.41
P9	customNV2.xls	-37.1653	-281.16	1047.76	12.99649	109.0754	-567.27
P9	customNV3.xls	-31.7445	-281.38	760.01	17.73475	104.8624	-613.68
P10	customNV1.xls	-48.9488	-235.16	768.86	21.1631	103.2015	-1353
P10	customNV2.xls	-56.8279	-242.49	909.8	21.91639	86.28221	-1217.43
P10	customNV3.xls	-47.7232	-343.92	873.72	15.50353	119.9417	-274.05
P11	customNV1.xls	-27.2885	-189.43	555.18	20.15267	93.18007	
P11	customNV2.xls	-16.425	-26.57	111.96	41.10109	54.46705	-597.11
P11	customNV3.xls	-48.988	-366.4	779.42	15.72213	90.9963	-725.35
P12	customNV1.xls	-56.3527	-457.96	858.46	15.82416	111.0549	-619.76
P12	customNV2.xls	-59.2569	-287.7	717.11	16.00021	113.2718	-594.7
P12	customNV3.xls	-53.6425	-256.92	687.15	12.48802	103.4264	-610.17
P13	customNV1.xls	-36.6092	-169.51	626.87	15.95688	103.6935	-221.06
P13	customNV2.xls	-45.4838	-166.94	626.48	13.4266	86.69813	
P13	customNV3.xls	-52.5275	-374.65	733.18	20.09357	120.5814	-577.19
P14	customNV1.xls	-38.5847	-249.53	241.74	16.10871	106.8948	-284.06
P14	customNV2.xls	-52.0901	-222.41	561.16	11.50333	111.9489	-217.45
P14	customNV3.xls	-62.7469	-429.85	815.08	11.62794	105.1543	-338.64
P15	customNV1.xls	-21.5613	-195.91	235.96	17.60464	87.9407	-215
P15	customNV2.xls	-11.2244	-117.03	438.13	23.01423	112.0763	-286.77
P15	customNV3.xls	-20.1427	-140.01	51.08	54.06987	90.1147	-330.62
P16	customNV1.xls	-35.699	-257.32	375.58	19.57399	73.60338	-698.45
P16	customNV2.xls	-27.4948	-195.17	461.53	15.0822	84.53019	-649.74
P16	customNV3.xls	-31.3548	-278.34	532.81	11.41119	92.07795	-603.55
P17	customNV1.xls	-30.7319	-272.85	483.38	23.63522	96.95841	-462.23
P17	customNV2.xls	-42.8581	-212.53	356.05	26.94761	67.53399	-400.34
P17	customNV3.xls	-8.82343	-104.5	253.67	13.24433	97.6494	-463.76
P18	customNV1.xls	-18.0812	-34.39	10.31	49.81928	53.36019	#VALUE!
P18	customNV2.xls	-39.8567	-199.59	619.84	7.732821	85.13908	-886.18
P18	customNV3.xls	-21.7476	-84.54	64.47	63.28925	62.62281	-386.95

impulseUT	mov-Init	UTT-vel-P	UTT-start	UTT-end
-27.0408	550	591	610	1306
-16.0232	354	388	406	1094
-7.32426	409	440	464	1188
-5.0177	403	469	496	1067
-6.57003	465	494	525	1049
-3.28292	482	510	528	1096
-7.29632	378	418	450	1029
-10.9062	361	392	408	1033
-10.8155	519	544	563	1089
0.66883	332	370	388	846
-18.3051	351	382	396	855
-43.9854	350	411	426	977
-21.7873	409	457	490	1162
-18.8449	431	479	503	1241
-19.4755	397	443	471	1203
-7.38493	396	430	440	1111
-19.7341	379	417	452	898
-2.79232	415	443	464	1167
-0.6296	393	454	467	1123
-9.04978	442	481	496	1259
-29.1654	444	489	505	946
-24.4558	357	450	469	1015
-35.9895	399	451	471	1129
-3.27191	448	496	510	1172
-16.0964	403	444	461	1023
-21.7142	361	398	420	1004
-26.3082	377	426	439	1024
-32.4807	385	429	444	947
-18.9633	331	368	383	1033
-32.0136	437	490	505	1233
-1.46419	243	263	267	1043
-68.6286	441	472	491	934
-29.9626	414	450	469	1116
-5.01523	399	439	463	1099
-17.5467	463	505	526	1176
-16.3277	373	422	437	1152
-6.51219	440	482	499	
-13.3025	451	485	501	1230
-17.2178	390	490	502	1015
-30.362	390	449	464	1023
-17.1551	350	385	404	1038
-7.65738	426	497	514	1049
-15.5398	454	483	501	1182
7.952057	161	258	262	1476
-0.73224	400	440	462	884
-34.4795	422	457	482	1036
-26.1919	488	524	541	1197
-34.5688	428	458	476	1068
-29.8205	443	478	499	1021
-46.4858	447	485	508	1066
-0.44105	269	308	312	918
-10.9903	393	449	466	856
-5.36866	211	254	258	1075
