# Supplementary material for: Moving from stable standing to single-limb stance or an up-on-the-toes position: The importance of vision to dynamic balance control
Source: PLoS One. 2024 Jul 23;19(7):e0307365. doi: 10.1371/journal.pone.0307365 (PMC11265682; doi:10.1371/journal.pone.0307365)
Supplement: S2 File — (PDF) [file pone.0307365.s002.pdf]

		CoP-dispA	impulseAP.	CoP-SD S	CoP-vel Pk	Pk CoP-ve	ave-CoP S	Pk CoP SS
P1	NDom1.xls	-69.3964	-27.2627	19.63962	-425.3	804.275	149.2578	185.1196
P1	NDom2.xls	-55.7484	-23.3541	17.10024	-368.65	494.425	146.1466	167.2256
P1	NDom3.xls	-63.3011	-26.538	14.63296	-424.65	724.4	137.7416	149.8279
P2	NDom1.xls	-78.1091	-45.4062	10.2571	-467.25	933.85	125.0001	133.2909
P2	NDom2.xls	-25.4672		11.36169	-364.4	647.65	131.6937	138.9108
P2	NDom3.xls	-82.7552	-51.9355	12.02074	-538.775	1008.275	134.7558	144.8308
P3	NDom1.xls	-50.5645	-40.7137	14.31485	-252.75	919.325	138.0852	152.3435
P3	NDom2.xls	-65.8876	-34.1322	14.76186	-422.15	853.6	140.472	153.4224
P3	NDom3.xls	-58.9869	-33.1708	12.21598	-305.4	880.15	137.5895	142.9791
P4	NDom1.xls	-50.095	-27.9264	14.33543	-397.85	402.675	150.7055	168.887
P4	NDom2.xls	-56.3055	-38.4536	12.86421	-456.025	896.35	145.0976	171.4455
P4	NDom3.xls	-38.6359	-32.5683	6.438566	-300.95	487.925	170.0586	181.6131
P5	NDom1.xls	-48.2368	-21.9881	11.0508	-271.3	546.9	115.4956	127.9142
P5	NDom2.xls	-31.8711	-22.4056	12.39697	-167.9	691.575	133.4949	145.0659
P5	NDom3.xls	-46.875	-24.439	22.83768	-156.6	721.7	159.1653	178.425
P6	NDom1.xls	-59.7761	-23.3572	14.09552	-296.1	1160.325	131.3656	153.8449
P6	NDom2.xls	-71.7148	-22.8472	14.82348	-471.325	1007.2	127.6323	149.4402
P6	NDom3.xls	-68.1482	-23.666	17.52922	-380.15	1021.625	139.3232	160.6158
P7	NDom1.xls	-29.4816	-15.0076	16.09644	-226.125	405.8	154.678	168.6554
P7	NDom2.xls	-41.1555	-17.7494	15.97906	-314.85	450.85	154.952	168.9525
P7	NDom3.xls	-44.6621	-17.6071	15.85575	-258.15	479.55	149.1262	163.6769
P8	NDom1.xls	-68.6391	-22.307	12.24186	-486.975	1427.275	130.1201	142.1539
P8	NDom2.xls	-39.8299	-14.8515	13.64677	-307.075	852.35	134.9437	161.7501
P8	NDom3.xls	-70.6618	-19.2787	13.67012	-423.275	907.65	130.372	152.3252
P9	NDom1.xls	-70.5881	-25.4958	14.67473	-498.025	933.225	138.4136	158.7109
P9	NDom2.xls	-47.2321	-23.4292	15.93036	-259.4	808.675	146.7755	164.6179
P9	NDom3.xls	-64.6299	-26.8495	16.31583	-282.7	1141.55	145.7816	169.8381
P10	NDom1.xls	-65.6599	-29.8594	26.2234	-318.775	999.225	140.9186	178.8631
P10	NDom2.xls	-42.8972	-24.786	15.17513	-214.2	513.375	149.5083	174.5468
P10	NDom3.xls	-55.9683	-27.3349	16.49657	-279.4	694.8	131.9547	156.0327
P11	NDom1.xls	-48.7541	-20.3823	17.84166	-360.175	523.15	132.3732	146.1719
P11	NDom2.xls	-51.6846	-26.4503	15.95626	-327.225	591.825	129.3344	142.3214
P11	NDom3.xls	-48.8973	-27.3866	20.04135	-291.575	636.925	138.6477	156.5997
P12	NDom1.xls	-80.1563	-33.6118	12.04307	-483.65	1259.25	112.4239	131.7947
P12	NDom2.xls	-52.2084	-29.8737	17.16845	-479.175	980.75	142.2799	165.3286
P12	NDom3.xls	-62.4284	-26.8556	12.77292	-419.725	879.75	110.5044	130.9476
P13	NDom1.xls	-60.9989	-19.5708	14.78214	-298.975	628.95	144.9153	159.1761
P13	NDom2.xls	-48.4858	-17.7012	9.688218	-293.75	628.625	114.7463	133.1212
P13	NDom3.xls	-65.4849	-19.9527	14.65608	-410.75	594.05	148.0299	162.3231
P14	NDom1.xls	-41.5112	-25.7657	16.47874	-329.875	815.3	147.0803	168.2088
P14	NDom2.xls	-60.4122	-22.6022	14.82932	-604.475	536.6	143.2904	164.0078
P14	NDom3.xls	-74.514	-25.6657	10.65073	-415.4	708.8	107.7157	126.627
P15	NDom1.xls	-36.5293	-17.003	19.72509	-263.4	868.1	157.642	179.9847
P15	NDom2.xls	-32.1796	-18.0865	17.24192	-121.475	684.325	150.9537	176.5214
P15	NDom3.xls	-25.6256	-9.38231	22.17078	-181.375	516.2	159.5917	182.9744
P16	NDom1.xls	-57.3801	-33.1783	19.6474	-485.1	911.4	159.2135	175.8949
P16	NDom2.xls	-60.396	-34.1475	18.46837	-261.85	778.2	164.7503	189.198
P16	NDom3.xls	-80.7951	-34.7242	18.05598	-646.175	928.1	160.1619	184.9119
P17	NDom1.xls	-60.637	-48.3767	10.60355	-320.575	714.65	119.3813	125.399
P17	NDom2.xls	-82.315	-51.3898	11.88891	-467.425	1052.4	137.1242	142.172
P17	NDom3.xls	-98.2683	-51.9052	9.668607	-672.35	1080.85	128.9371	134.3017
P18	NDom1.xls	-72.4267	-15.0726	11.4979	-475.2	914.95	106.6628	120.0793
P18	NDom2.xls	-106.336	-18.9624	9.882609		1498.375	110.7613	122.2283
P18	NDom3.xls	-87.8317	-20.2691	14.27095	-575	1305.225	139.8115	153.9403

SD CoP-ve	Pk CoP ret	mov-Init	end-Init	timeAPA	begin_SS	time to SS	end_SS
162.7786	-717.35	344	393	0.49	422	0.78	717
86.55412	-308.85	289	336	0.47	380	0.91	785
120.6688	-703.2	335	385	0.5	419	0.84	719
130.8451	-738.1	302	346	0.44	370	0.68	648
117.8712	-599.7	277	377	1	403		693
117.3663	-606.4	421	467	0.46	490	0.69	792
119.3761	-507.8	363	412	0.49	433	0.7	773
118.6247	-503.9	388	431	0.43	456	0.68	796
115.3191	-626.3	424	471	0.47	491	0.67	821
247.7044	-2484.28	377	422	0.45	484	1.07	813
113.3943	-591.925	401	447	0.46	473	0.72	835
62.3418		315	363	0.48	410	0.95	
57.95142	-298.9	436	482	0.46	511	0.75	908
86.15148	-410.525	413	477	0.64	507	0.94	894
85.36663	-258.6	436	481	0.45	511	0.75	873
193.51	-1743	439	485	0.46	506	0.67	824
75.76823		330	372	0.42	394	0.64	
170.2991	-1392	448	497	0.49	525	0.77	843
122.6503	-679.775	345	400	0.55	451	1.06	797
130.2704	-843.275	425	475	0.5	522	0.97	852
99.00565	-504.075	460	508	0.48	552	0.92	917
220.0431	-1373.48	404	447	0.43	465	0.61	648
144.8586	-1100.95	410	446	0.36	473	0.63	802
125.0668	-909.875	417	460	0.43	481	0.64	802
101.7668	-438.525	454	501	0.47	522	0.68	896
114.3096	-517.025	458	509	0.51	537	0.79	913
161.1022	-1257.43	455	501	0.46	520	0.65	878
138.9908	-574	346	395	0.49	416	0.7	771
160.5491	-1116.45	430	486	0.56	530	1	870
121.5339	-525.575	388	438	0.5	472	0.84	801
81.92368	-342.65	419	464	0.45	503	0.84	804
73.19701	-261.875	444	493	0.49	525	0.81	853
63.99864	-191.8	412	464	0.52	496	0.84	844
114.7371	-503.35	450	494	0.44	512	0.62	813
123.4941	-611.275	395	440	0.45	462	0.67	849
107.2363	-568.825	374	419	0.45	435	0.61	757
116.0103	-640.975	419	469	0.5	508	0.89	853
100.8349	-648.85	395	444	0.49	474	0.79	802
115.9777	-805.425	460	506	0.46	543	0.83	937
125.4449	-495.375	429	481	0.52	511	0.82	812
164.2999	-956.825	399	448	0.49	490	0.91	768
84.76248	-327.425	428	472	0.44	494	0.66	782
124.6252	-617.25	463	515	0.52	551	0.88	902
113.4197	-580	427	475	0.48	513	0.86	879
92.46093	-373.075	473	514	0.41	578	1.05	1001
157.8248	-674.25	419	469	0.5	499	0.8	759
147.0074	-618.725	485	546	0.61	578	0.93	919
142.8813	-575.025	469	515	0.46	542	0.73	890
94.72572	-494.725	412	467	0.55	491	0.79	767
137.4656	-983.35	409	457	0.48	477	0.68	790
111.4132	-896.5	401	444	0.43	464	0.63	843
109.2407	-1093.43	266	306	0.4	324	0.58	669
85.9744	-940.575	425	460	0.35	475	0.5	827
136.2297	-1082.7	406	447	0.41	465	0.59	830
